# Zein/Shellac Composite Films with Limonin and Resveratrol: Characterization and Application for Strawberry Preservation

**DOI:** 10.3390/foods15010083

**Published:** 2025-12-26

**Authors:** Limin Wang, Qianfei Jia, Yuxi Qin, Shufen Li, Zijian Wu

**Affiliations:** 1 College of Biotechnology and Food Science, Tianjin University of Commerce, Tianjin 300134, China; wlm0218@tjcu.edu.cn (L.W.); fqj428531@163.com (Q.J.); 13343200305@163.com (Y.Q.); 13609905179@163.com (S.L.); 2 Tianjin Key Laboratory of Food Biotechnology, Tianjin University of Commerce, Tianjin 300134, China; 3 Key Lab of Agricultural Products Low Carbon Cold Chain, Ministry of Agriculture and Rural Affairs, Tianjin 300134, China

**Keywords:** zein/shellac composite films, resveratrol, limonin, antioxidative, strawberry preservation

## Abstract

This study aimed to fabricate an active film incorporating limonin (LM) and resveratrol (RES) within a zein/shellac (ZS) matrix for use in strawberry preservation. Zein/shellac composite films embedded with varying concentrations of LM (0–4% *w*/*w*) and RES were successfully fabricated using coaxial electrospinning. The prepared films were comprehensively characterized for their mechanical properties, water vapor permeability (WVP), antioxidant capacity (DPPH, ABTS, FRAP), and antibacterial efficacy against *E. coli* and *S. aureus*. Mechanical properties and WVP results revealed that the ZSLM4R film exhibited an elongation at break (EBA) of 28.91%, tensile strength (TS) of 0.93 MPa, elastic modulus (EM) of 40.76 MPa, and a WVP of 1.55 g mm/m^2^. h. kPa. Furthermore, LM and RES increased the antioxidant properties of the composite film. ZSLM4R’s free radical scavenging activities against DPPH and ABTS were 68.14% and 89.69%, respectively. The composite film also demonstrated strong antibacterial efficacy against *E. coli* and *S. aureus*. When applied to strawberries, ZSLM4R packaging effectively extended the fruit’s shelf life compared to the unwrapped and commercial polyethylene (PE) controls. These obtained results indicate that LM/RES-incorporated zein/shellac composites are a promising eco-friendly packaging alternative for preserving perishable fresh produce and extending its shelf life.

## 1. Introduction

Food packaging serves a critical role in product protection throughout transportation and storage [1]. Currently, petroleum-based synthetic polymers are widely used to produce food packaging owing to their cost-effectiveness, lightweight nature, stability, and ease of processing [2]. However, these materials significantly contribute to environmental pollution and biosecurity risks. Consequently, plant-derived protein-based films have gained attention as a promising, eco-friendly alternative for quality preservation in sustainable food packaging. Single plant protein-based films may exhibit poor mechanical properties and weak water resistance properties. To enhance this property of such films, a variety of natural biopolymers (e.g., proteins, starches, and resins) are increasingly utilized. Our previous study fabricated a zein/shellac composite film via coaxial electrospinning, utilizing shellac, a biopolymer material, which interacted with zein via hydrophobic interactions. This formed a film that exhibited excellent mechanical properties and demonstrated effective barriers to both water vapor and oxygen [3]. However, a major challenge for these biopolymer-based food packaging films is their inability to actively address food spoilage. While it offers a physical barrier, it lacks sufficient antimicrobial and antioxidant capabilities. Therefore, active packaging incorporating functional additives (e.g., antimicrobials and antioxidants) is essential to bridge this gap.

Currently, the benefits of active protein-based films are widely recognized. For instance, Li et al. [4] utilized a zein-based film loaded with resveratrol (RES) for pork preservation, and Xu et al. [5] applied a zein–quercetin composite coating for strawberry preservation. They both efficiently maintained the nutrient quality and acceptable appearance of products, prolonging the shelf life of foodstuff. These studies demonstrated the potential of zein-based active film. However, the development and application of high-performance composite films for perishable fruits still face hurdles. Relying on single active components often fails to meet the requirements for superior moisture barriers and sustained antimicrobial capacity. Thus, a combination of the high-barrier zein/shellac matrix with a synergistic functional additive may be necessary to address these challenges.

Limonin (LM), a highly oxidized tetracyclic triterpenoid, extensively exists in citrus peels and seeds [6,7]. LM exhibits diverse biological properties, including anti-inflammatory, anti-apoptosis, and significant antibacterial effects on some fungi, such as *Penicillium italicum* and *Rhizopus* [8,9,10]. These attribute LM as a promising antimicrobial component for bioactive food packaging. However, its relatively weak antioxidant capacity limits its effectiveness against oxidative spoilage. To address this limitation, we incorporated resveratrol (RES)—a bioactive polyphenol abundant in grapes, peanuts, and other plants. RES possesses multiple phenolic hydroxyl groups that enable potent antioxidant activity through interactions with biological macromolecules, along with anti-inflammatory and anti-cancer functions [11]. Its efficacy in enhancing food preservation has been demonstrated in edible films; Yuan et al. [12] significantly enhanced the antioxidant capacity of loquat seed starch films by adding RES. Thus, the synergistic combination of LM and RES has the potential to provide comprehensive antimicrobial and antioxidant protection within the zein/shellac composite film.

As a fabrication technique, electrospinning offers distinct advantages for creating nanofibrous materials with tailored morphological characteristics, including high surface-to-volume ratios and adjustable porosity. These properties make it promising for applications like active food packaging and drug delivery. Compared with conventional blend electrospinning, coaxial electrospinning encapsulates active compounds within a shell layer, reducing burst release and slowing rapid consumption. Studies showed that coaxial nanofibers exhibit slower release rates and different kinetics compared to uniaxial fibers [13]. The core–shell structure also delays active compound diffusion into food simulants more effectively than uniaxial fibers [14].

This study developed an LM/RES-embedded zein/shellac composite film through coaxial electrospinning. Initially, this work investigated the formation and core–shell structure of the composite film by SEM and FTIR. Then, it characterized its physicochemical, mechanical, and barrier properties. Additionally, the synergistic effect of LM and RES on enhancing the film’s in vitro antioxidant and antibacterial activities was evaluated. Finally, the active composite film’s potential for extending the shelf life of fresh strawberries was assessed by monitoring weight loss, firmness, TSS, TA, and MDA during storage.

## 2. Materials and Methods

### 2.1. Materials

Limonin, resveratrol, zein (BR grade and 92% purity), and shellac were sourced from Shanghai Yuanye Biotechnology Co., Ltd. (Shanghai, China). Polyvinylpyrrolidone (PVP, K88-96, MV 1,300,000) and N, N-Dimethylformamide (DMF) were purchased from Aladdin Bio-Technology (Shanghai, China). All experiments employed analytical-grade reagents and deionized water. For antimicrobial assessment, *Staphylococcus aureus* (*S. aureus*) (ATCC 6538, Gram-positive) and *Escherichia coli* (*E. coli*) (ATCC 25922, Gram-negative) strains were acquired from Shanghai Luwei Technology Co., Ltd. (Shanghai, China).

### 2.2. Spinning Solutions Preparation

For the spinning solutions of coaxial electrospinning, the shell solution consisted of 10% *w/v* PVP in 85% ethanol (1 g PVP in 10 mL). The core solution of 21:4 *w*/*w* ratio of zein/shellac was prepared based on our previous study [3]. A certain mass of LM and RES was dissolved into DMF to prepare 0, 10, 20, and 40 mg/mL of LM reserve solution and 30 mg/mL of RES reserve solution, respectively. A mixed reserve solution containing 40 mg/mL LM and 30 mg/mL RES was also prepared. After that, the core solution was obtained by mixing the zein/shellac solution with the above reserve solution in a ratio of 4:1. Eventually, spinning solutions were obtained with LM concentrations of 0%, 1%, 2%, and 4% *w*/*w* and a RES concentration of 3%, which were named ZS, ZSLM1, ZSLM2, ZSLM4, ZSLM4R, and ZSR, respectively (Table 1).

### 2.3. Fabrication of Composite Films

The film preparation was conducted on a YFSP-T electrospinning system (Yunfan Science and Technology Co., Ltd., Tianjin, China) with coaxial configuration. The core and shell solutions were separately loaded into 5 mL syringes equipped with a coaxial spinneret (inner needle: 0.6 mm; outer needle: 1.4 mm). The following electrospinning parameters were applied: the core solution flow rate was maintained at 2 mL/h, and the shell solution at 3 mL/h, and an applied potential difference of 10 kV (positive) to 2 kV (negative). The spinneret-to-collector distance was fixed at 15 cm. Throughout the experiments, the electrostatic spinning was operated under controlled environmental conditions (25 ± 5 °C; 30 ± 5% RH).

### 2.4. Characterization of Composite Films

#### 2.4.1. Scanning Electron Microscopy (SEM)

The surface morphology of composite films was observed using scanning electron microscopy (Phenom XL, Phenom-World B.V., Eindhoven, The Netherlands) at an acceleration voltage of 10 kV.

#### 2.4.2. Fourier-Transform Infrared Spectroscopy (FTIR)

The FTIR characterization of the composite films was acquired using a spectrometer (Lumos, Brucker, Germany). Spectra were collected according to a modified method based on Li et al. [1], with parameters set to 4 cm^−1^ resolution across the 600–4000 cm^−1^ wavenumber range.

#### 2.4.3. X-Ray Diffraction (XRD) Analysis

The XRD analysis of the composite films was conducted by an X-ray diffractometer (Ultima IV, Rigaku Corporation, Tokyo, Japan) equipped with Cu-kα radiation. Scans were performed across a 2θ range of 5–60° with a scanning rate of 5°/min.

#### 2.4.4. Mechanical Properties

Mechanical characterization of the composite films was performed using an AGS-X electronic universal testing machine according to the method of Hasan et al. [15]. The composite films were cut to a size of 50 mm × 20 mm, and a force of 20 N load was employed. The measurement was performed at a speed of 5 mm/s. The elongation at break (EAB), tensile strength (TS), and elastic modulus (EM) were determined from six replicate measurements for each sample. The thickness of the composite film was measured by an electronic digital micrometer.

#### 2.4.5. Water Vapor Permeability (WVP)

The WVP values of the composite films were measured using a previous approach with some minor changes [16]. In brief, 5 g of anhydrous CaCl_2_ was loaded into weighing bottles (40 mm × 25 mm). Composite film samples (5 × 5 cm) were securely sealed onto the bottle mouths. Then, they were transferred to a controlled environment dryer (Tokyo Rikakikai Co., Ltd., Tokyo, Japan) and maintained for 4 days at 25 °C and 75% RH. The changes in bottle weights were measured at 4 h intervals. The WVP was calculated as Equation (1):

(1) $$\begin{array}{c} WVP=\left(\Delta m\times L\right)/(\Delta t\times A\times \Delta P) \end{array}$$

where Δm means the weight change (g), *L* is average film thickness (mm), Δ*t* denotes the time interval for weight gain (s), *A* denotes the effective film permeation area (m^2^), and ΔP is the water vapor pressure difference (kPa) calculated from RH and temperature across both sides of the films.

### 2.5. Antioxidant Properties

#### 2.5.1. DPPH Radical Scavenging Activity

The DPPH radical scavenging activity was conducted using a slightly modified method [17]. A total of 5 mg of composite film samples were dissolved in 2 mL of 0.1 mM DPPH solution and incubated in darkness at ambient temperature for 30 min. The absorbance measurements at 517 nm were performed using an Infinite M200 Pro microplate reader (Tecan Ltd., Männedorf,, Switzerland). Radical scavenging activity was determined according to Equation (2):

(2) $$\begin{array}{c} DPPH\;radical\;scavenging\;activity\;(\%)=\frac{A_{0}-A_{1}}{A_{0}}\times 100 \end{array}$$

where *A*_1_ and *A*_0_ are the absorbance values at 517 nm for DPPH solutions with and without the sample, respectively.

#### 2.5.2. ABTS Radical Scavenging Activity

ABTS radical scavenging activity of composite film samples was evaluated according to the method with some modifications [18]. Briefly, ABTS solution was obtained by mixing equal volumes of 7.4 mM ABTS and 2.6 mM potassium persulfate, followed by 12 h incubation in darkness at 25 °C. The solution was then adjusted with ethanol to achieve an absorbance of 0.70 ± 0.01 at 734 nm. A total of 2 mg of composite films was dissolved in 5 mL of ABTS solution, and the solution was mixed and kept in the dark for 30 min. The absorbance of the resulting solution at 734 nm was measured by a microplate reader. The ABTS radical scavenging activity was assessed by Equation (3):

(3) $$\begin{array}{c} ABTS\;radical\;scavenging\;activity\;(\%)=\frac{A_{0}-A_{1}}{A_{1}}\times 100 \end{array}$$

where *A*_1_ and *A*_0_ are the absorbance values at 734 nm for ABTS solutions with and without the sample, respectively.

#### 2.5.3. The Ferric Reducing Ability Power (FRAP)

The ferric reducing antioxidant power (FRAP) was assessed following the modified method [19]. The FRAP working solution was prepared by mixing 0.3 M acetate buffer (pH 3.6), 10 mM TPTZ (2,4,6-tripyridyl-s-triazine) dissolved in 40 mM HCl, and 20 mM FeCl_3_ solution in a 10:1:1 volume ratio. Then, 5 mg of the composite film samples were added to 2 mL of FRAP reagent solution; after completing incubation for 30 min (37 °C, 5 min), the absorbance of the reaction mixture (FRAP supernatant) was measured at 593 nm. The solution without the composite film sample served as a control.

### 2.6. Antibacterial Properties

The antibacterial activities of composite film were assessed against Gram-negative *E. coli* and Gram-positive *S. aureus* using the plate counting method. A total of 60 mg of composite films were put into the centrifuge tubes with 1 mL of bacterial suspension (10^6^~10^7^ CFU/mL) and incubated in a constant temperature shaker for 5 h at 37 °C. The centrifuge tube without composite samples serves as a blank control. Then, 100 μL of the diluted bacterial solution was evenly applied to the LB plate. The plates were then incubated at 37 °C for 12 h. Photographs were taken and recorded. The number of bacterial colonies was calculated (the number of colonies was between 30 and 300). Equation (4) was used to calculate the bacteriostatic rate:

(4) $$\begin{array}{c} Antibacterial\;Rate\;\left(\%\right)=\frac{B-C}{B}\times 100\% \end{array}$$

where *B* and *C* are the number of colonies in the control group and the experimental group, respectively.

### 2.7. Strawberry Preservation

A batch of fresh strawberries (Dandong 99) was purchased from the local supermarket. Strawberries of uniform size, color, and ripeness and free from damage were selected and randomly divided into preservation groups (*n* = 10 per treatment). The strawberries were placed in a crisper, with the composite film covering the top, while an unwrapped group served as the blank control. The preservation experiments were conducted under ambient conditions (25 ± 2 °C, 60–70% RH) for 8 days, including the following groups: blank control group, PE group, ZS, ZSLM4, ZSLM4R, and ZSR composite film. On each sampling day (Day 0, 2, 4, 6, 8), three representative strawberries were randomly selected from one package that was opened for that specific day’s analysis (e.g., weight loss, firmness, and total acid).

#### 2.7.1. Weight Loss Measurement

The weights of the strawberries were measured every two days, and their weight loss was calculated relative to the initial weight of the strawberries according to Equation (5) [20]:

(5) $$\begin{array}{c} Weight\;loss\;(\%)=\frac{W_{0}-W_{t}}{W_{0}}\times 100 \end{array}$$

where *W*_0_ is the initial weight of the strawberry (g), and *W_t_* is the weight of the strawberry sample during storage (g).

#### 2.7.2. Hardness Measurement

Strawberry hardness was determined using a TA-XT Plus texture analyzer (Xiamen Chaoji Instrument and Equipment Co., Ltd., Xiamen, China) with a 2 mm diameter cylindrical probe. Measurements were conducted at a penetration depth of 10.0 mm with a constant speed of 1.0 mm/s. Five replicate tests were performed along the equatorial zone of each strawberry, and the average value was calculated to represent sample hardness.

#### 2.7.3. TSS and TA of Strawberries

Determination of total soluble solids (TSSs) and total acid (TA) of strawberries was carried out by a PAL-BX/ACID1 saccharometer (ATAGO Co., Ltd., Tokyo, Japan). A centrifuge was used to centrifuge at 6076× *g* for 10 min at 4 °C. A certain amount of supernatant was aspirated for the determination and diluted 50 times to read the acidity values, which are expressed as °Brix for soluble solids and % for total acid. The above experiments were repeated three times.

#### 2.7.4. MDA Content of Strawberries

Malondialdehyde (MDA) content was quantified following an adapted method of Qin et al. [21]. Strawberry tissue (1 g) was homogenized with 5 mL of 10% trichloroacetic acid (TCA), followed by centrifugation (8000× *g*, 10 min, 4 °C) to obtain the supernatant. Subsequently, 2 mL of supernatant and 2 mL of 0.67% thiobarbituric acid (TBA) solution (dissolved in 0.05 mol/L NaOH solution) were thoroughly mixed. The mixture was heated in boiling water (15 min), cooled (4 °C, 20 min), and centrifuged (8000× *g*, 15 min, 4 °C). Absorbance was measured at 450, 532, and 600 nm, and MDA content was calculated using Equations (6) and (7).

(6) $$\begin{array}{c} c(\mu mol/L)=6.45\left({OD}_{532}-{OD}_{600}\right)-0.56\times {OD}_{450} \end{array}$$

(7) $$\begin{array}{c} Total\;MDA\left(nmol/g\;m_{F}\right)=\frac{c\times V}{V_{s}\times m} \end{array}$$

where *c* is the MDA concentration in the reaction mixture; OD_450_, OD_532_, and OD_600_ are the absorbance values at 450 nm, 532 nm, and 600 nm, respectively; *V* (mL) is the total volume of the sample extraction supernatant; *V_s_* (mL) is the volume of extract taken during the determination; and *m* (g) is the strawberry pulp mass.

### 2.8. Statistical Analysis

All experiments were conducted in triplicate. Data are presented as mean ± standard deviation (SD). Statistical significance was determined by one-way analysis of variance (ANOVA) followed by Tukey’s post hoc test using IBM SPSS Statistics (Version 21.0; IBM Corp., Armonk, NY, USA). The graphs were created with OriginPro 2021 (OriginLab Corp., Northampton, MA, USA).

## 3. Results

### 3.1. Characterization of Zein/Shellac Composite Films Loaded with LM and RES

#### 3.1.1. Morphological Properties

**Figure 1:**
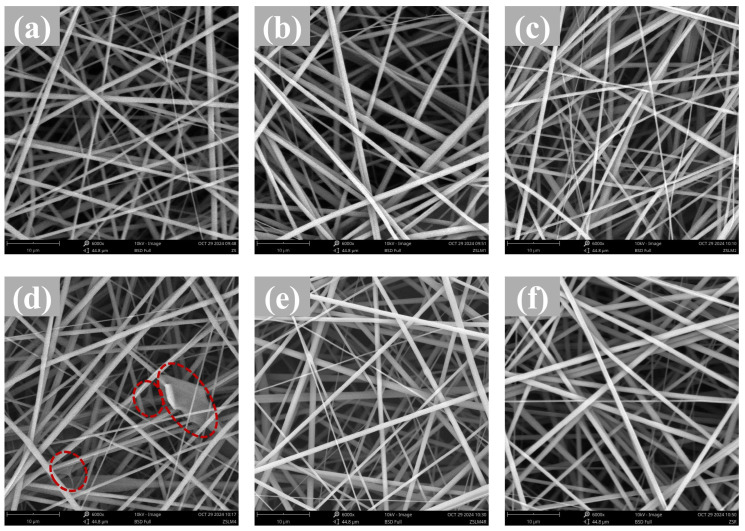
SEM images of the zein/shellac composite films loaded with LM and RES: (**a**) ZS; (**b**) ZSLM1; (**c**) ZSLM2; (**d**) ZSLM4; (**e**) ZSLM4R; (**f**) ZSR.

The morphology properties of zein/shellac composite films loaded with LM and RES were observed using scanning electron microscopy (SEM). The control zein/shellac composite films exhibited a smooth, bead-free, and homogeneous morphology (Figure 1a), indicating excellent compatibility of LM/RES in the zein/shellac matrix. The smooth and continuous fibers were also obtained in the ZSLM1 and ZSLM2 after LM addition, and these fibers were randomly distributed (Figure 1b,c). However, the fibers displayed an irregular and bead-like structure with a higher content LM loading (ZSLM4) (red dashed line in Figure 1d). It could be attributed to the increase in viscosity of the spinning solution at higher LM concentrations, which destabilizes the electrospinning jet during fiber formation [22]. Furthermore, RES-incorporated films (ZSLM4R) preserved uniformly dispersed fibers with smooth surfaces and non-bead structures, bead-free surfaces (Figure 1e), demonstrating that the incorporation of RES facilitated the production of uniformly dispersed fibers for composite film.

#### 3.1.2. Fourier-Transform Infrared Spectroscopy (FTIR) Analysis

**Figure 2:**
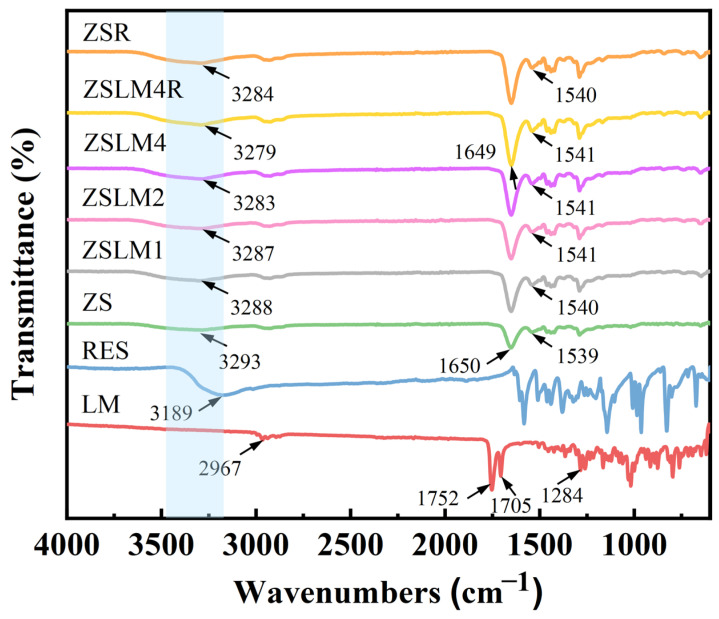
FTIR spectra of the zein/shellac composite films loaded with LM and RES.

The FTIR spectrum of zein/shellac composite films loaded with LM and RES is shown in Figure 2. The characteristic absorption peaks of LM mainly include 2967, 1752, 1705, and 1284 cm^−1^; the peak at 2967 cm^−1^ is attributed to the stretching vibration of =C-H groups.

The bands observed at both 1752 cm^−1^ and 1705 cm^−1^ are characteristic of C=O stretching vibrations. The absorption peak at 1284 cm^−1^ is assigned to the stretching vibration of the C-O group [8]. RES displayed a peak at 3189 cm^−1^, corresponding to -OH stretching [23]. In the zein/shellac composite film, the peak caused by -OH stretching vibration appeared at 3293 cm^−1^, while this peak shifts after LM addition; even the absorption peak of ZSLM4 was shifted to 3283 cm^−1^, indicating that there may be hydrogen-bonding interactions between LM and zein/shellac [4]. Similarly, RES incorporation (ZSLM4R and ZSR) caused further shifts in the -OH peaks (from 3282 cm^−1^ to 3279 cm^−1^ and from 3293 cm^−1^ to 3284 cm^−1^, respectively), suggesting that hydrogen bonding interactions may also exist between RES and zein/shellac.

For the ZS composite film, the characteristic peaks of LM and RES (600–1600 cm^−1^) disappeared after the formation of composite films, which indicates that LM and RES are successfully encapsulated in the zein/shellac composite fibers. In addition, the peaks at 1650 cm^−1^ (amide I, C=O stretching) and 1539 cm^−1^ (amide II, N-H bending) were observed for ZS composite film [24]. After LM and RES incorporation, while the amide I band of the composite film (ZSLM4R and ZSR) was not shifted compared with the ZS composite film, the intensity of the peaks increased gradually. Moreover, the amide II band of ZSLM4R and ZSR shifted to 1541 cm^−1^ and 1540 cm^−1^, respectively, which suggests that electrostatic interactions existed between zein/shellac, LM, and RES [25].

#### 3.1.3. X-Ray Diffraction (XRD) analysis

**Figure 3:**
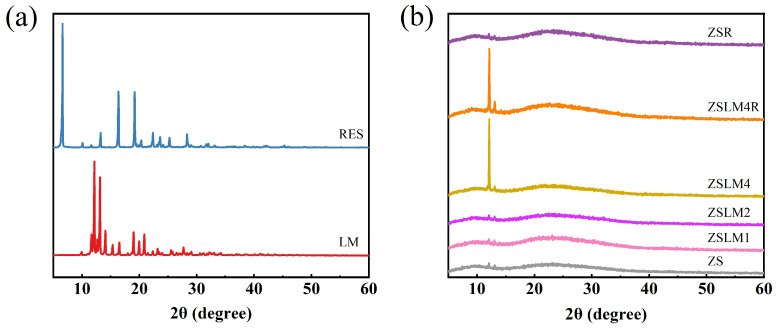
XRD pattern of (**a**) pure LM and RES; (**b**) zein/shellac composite films loaded with LM and RES.

XRD is commonly used to analyze crystalline materials for physical phase identification. As illustrated in Figure 3a, pure LM and RES exhibited characteristically intense peaks in the range of 2θ = 5–30°, demonstrating their crystalline properties; this result was consistent with previous research [26]. In addition, in the XRD patterns of the zein/shellac composite films (ZS, ZSLM1, ZSLM2, and ZSR) (Figure 3b), the sharp, small peaks characteristic of LM and RES were no longer observed, indicating that LM and RES were excellently compatible within the zein/shellac matrix [27]. Furthermore, as the amount of LM increased in zein/shellac composite films (ZSLM4), the XRD intensity of the characteristic peak also increased, indicating that the abundant LM in the ZS composite film matrix may have promoted molecular rearrangement, thereby increasing the ordered structure of the polymer molecules [28]. A similar phenomenon was also observed with RES incorporation in LM-loaded zein/shellac composite films (ZSLM4R).

#### 3.1.4. Mechanical Properties Analysis

**Figure 4:**
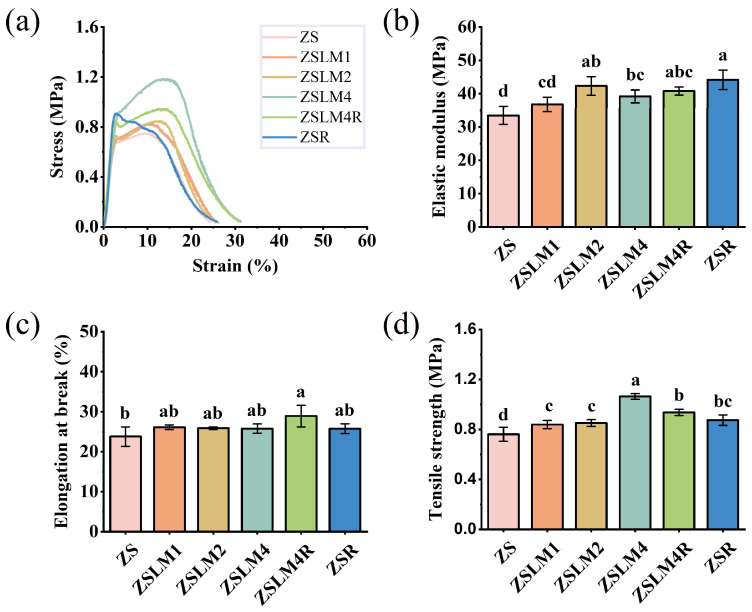
Mechanical properties of the zein/shellac composite films loaded with LM and RES: (**a**) typical stress–strain curves; (**b**) elastic modulus; (**c**) elongation at break; (**d**) tensile strength. Data are given as mean values ± SD. Different letters on the top of the data bars indicate significant differences (*p* < 0.05) between mean values.

The mechanical properties of films were crucial for protecting food during transportation, storage, and processing [29]. As shown in Figure 4, tensile strength (TS), elongation at break (EBA), and elastic modulus (EM) of the composite films were evaluated to assess their strength, ductility, and rigidity. It can be found that the stress–strain curves (Figure 4a) of the composite films are all plastic deformations. And notably, LM incorporation enhanced the mechanical properties of the composite film; at the highest LM loading (ZSLM4), EM (39.19 MPa) (Figure 4b), EBA (25.75%) (Figure 4c), and TS (1.06 MPa) (Figure 4d) increased moderately compared to control ZS films. Specifically, while ZSLM2 (41.80 MPa) and ZSR (40.20 MPa) exhibited the highest EM values, the EM of ZSLM4 was still significantly higher than that of the control ZS film. This suggested that the incorporation of LM improved the mechanical properties of composite film to a certain degree, which might be related to the hydrogen bonds between LM and compounds in zein/shellac film [5]. As for RES addition, the EAB and EM value of the composite film (ZSLM4R) had no significant change, but the TS reduced to 0.93 MPa. This might be attributed to the introduction of RES slightly disrupting the ZSLM4 network structure [11]. A similar phenomenon was observed in a previous study [30], where polyphenol extracts from purple rice (PRE) disrupted the matrix network of film, thereby reducing the TS value.

#### 3.1.5. Water Vapor Permeability (WVP) Analysis

**Figure 5:**
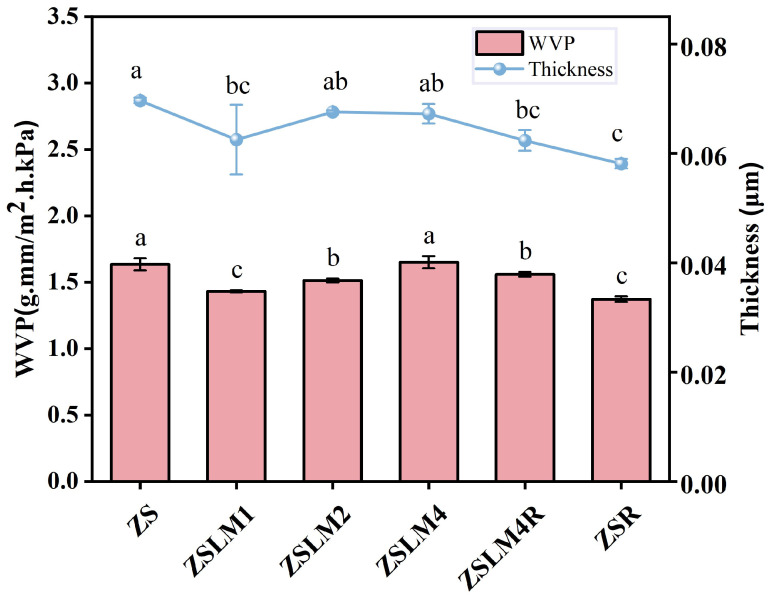
The WVP and thickness of the zein/shellac composite films loaded with LM and RES. Data are given as mean values ± SD. Different letters on the top of the data bars indicate significant differences (*p* < 0.05) between mean values.

Water vapor permeability (WVP) critically determines packaging efficacy in moisture-sensitive food applications. As shown in Figure 5, the control ZS film exhibited a WVP of 1.63 g mm/m^2^. h. kPa. After adding 1% *w*/*w* LM (ZSLM1) to the ZS composite film, the WVP value of films was significantly decreased to 1.43 g mm/m^2^. h. kPa (*p* < 0.05). However, when the concentration of LM further increased (ZSLM1-ZSLM4), the WVP value of these composite films gradually increased (from 1.43 to 1.65 g mm/m^2^. h. kPa.); this may be due to the slightly increased thickness of composite films (ZSLM1-ZSLM4) caused by LM addition, which was consistent with our previous findings [3]. Additionally, excessive incorporation of hydrophobic compounds can disrupt the polymer matrix network, as reported by He et al. [31], potentially contributing to the permeability increase. In addition, the WVP of the ZSLM4R was slightly decreased to 1.55 g mm/m^2^. h. kPa with RES incorporation. This decrease could be related to the twisted path of water molecules that was created with the cross-linked networks of RES and compounds in composite film [32,33]. In summary, the ZSLM4R composite films demonstrate optimized moisture barrier properties, which are potentially suitable for food preservation.

### 3.2. The Antioxidant Properties of Zein/Shellac Composite Films Loaded with LM and RES

**Figure 6:**
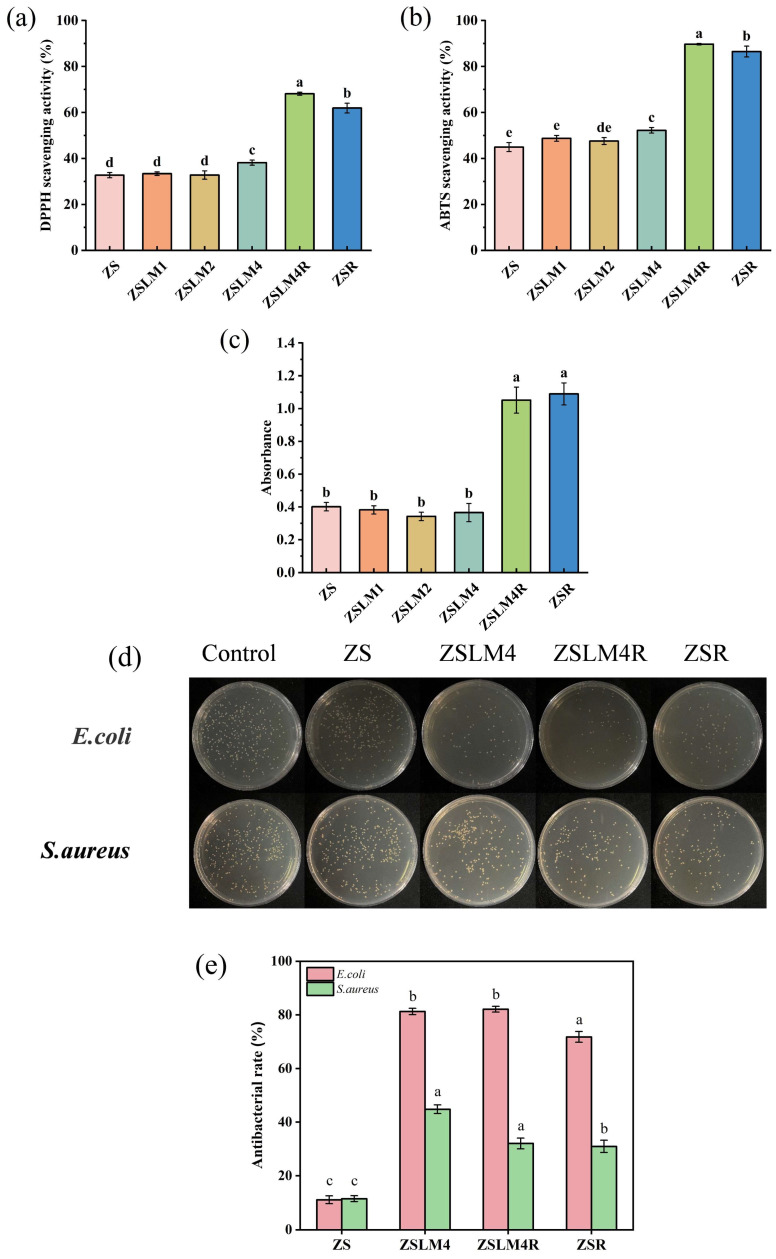
(**a**) The DPPH, (**b**) ABTS radical scavenging activity, and (**c**) FRAP of zein/shellac composite films loaded with LM and RES; (**d**) antibacterial activity and (**e**) antibacterial rates of *E. coli* and *S. aureus* by zein/shellac composite films loaded with LM and RES. Data are given as mean values ± SD. Different letters on the top of the data bars indicate significant differences (*p* < 0.05) between mean values.

The antioxidant capacity of packaging films is critical for preventing oxidative food spoilage [34,35]. We evaluated this property through DPPH, ABTS radical scavenging, and ferric reducing antioxidant power (FRAP) assay. As shown in Figure 6a,b, control zein/shellac (ZS) composite films exhibited minimal DPPH and ABTS radical scavenging activities, which is in agreement with the previous study about the low antioxidant properties of shellac [12]. The incorporation of LM into the composite films showed a concentration-dependent effect on antioxidant capacity. Specifically, ZSLM4 exhibited a significant increase in DPPH (38.18%) and ABTS scavenging activity (52.23%) compared to the control ZS film (DPPH: 35.51%, ABTS: 47.78%) (*p* < 0.05). ZSLM1 and ZSLM2 showed no statistically significant difference in antioxidant activity compared to ZS (Figure 6a,b). This is consistent with the previous reports of LM’s relatively weak antioxidant capacity [36]. Moreover, when the RES was incorporated into a zein/shellac composite film (ZSR), the DPPH and ABTS scavenging capacity significantly increased from 38.18% to 61.92% and 52.23% to 86.49%, respectively (*p* < 0.05). This is likely due to RES’s three phenolic hydroxyl groups that could destroy reactive oxygen and provide hydrogen atoms to DPPH and ABTS, thereby increasing their radical scavenging ability [37]. Notably, LM/RES dual-additive composite films (ZSLM4R) exhibited the highest DPPH (68.14%) and ABTS (89.70%) free radical scavenging activity, respectively, which indicated that LM and RES possessed a synergistic antioxidant effect to a certain degree.

FRAP assay results (Figure 6c) showed a similar trend observed in DPPH and ABTS radical scavenging assays. While LM incorporation (ZSLM1-ZSLM4) did not significantly improve the FRAP values compared to ZS film, the addition of RES (ZSR and ZSLM4R) caused a substantial and significant increase (*p* < 0.05) from 0.13 to 1.67 and 1.62, respectively. The above results demonstrate that RES incorporation dramatically enhances both the radical scavenging capacity and ferric reducing power of the zein/shellac composite films; LM incorporation provides only improvement in radical scavenging and no significant effect on FRAP; and the combination of LM and RES exhibits a degree of synergistic interaction, yielding the highest overall antioxidant activity. This significantly enhanced antioxidant activity has the potential to extend food shelf life by mitigating oxidative degradation in active packaging applications.

### 3.3. Antibacterial Properties of Zein/Shellac Composite Films Loaded with LM and RES

The antibacterial efficacy of composite films was evaluated against Gram-negative bacterium *E. coli* and Gram-positive bacterium *S. aureus*. Figure 6d depicts the colony results of *E. coli* and *S. aureus* after incubation with various composite films, and Figure 6e corresponds to their antibacterial rate. The control ZS films exhibited no significant antibacterial activity against either *S. aureus* or *E. coli* compared to blank controls (incubation without films), consistent with previous findings that zein and shellac lack inherent antimicrobial properties [38,39]. In addition, it can be seen that incorporation of either LM alone (ZSLM4 film) or RES alone (ZSR film) conferred antimicrobial activity against both bacterial strains (*p* < 0.05) (Figure 6e). This observation aligns with research on the individual antibacterial properties of LM and RES [40,41]. Notably, the dual-additive composite film (ZSLM4R) demonstrated relatively excellent antibacterial efficacy against *E. coli* among all the groups treated with composite film, and its effect against *S. aureus* was weaker than that of *E. coli*. This phenomenon was in line with the results of Abdalbeygi et al. [42], who demonstrated that RES has a better inhibitory effect against *S. aureus*. These results highlight the promising antimicrobial potential of the ZSLM4R composite film, particularly for food packaging applications requiring broad-spectrum protection, such as extending the shelf life of highly perishable commodities like strawberries, which are susceptible to spoilage by both bacterial types.

### 3.4. Preservation of Strawberries Using Zein/Shellac Composite Films Loaded with LM and RES

#### 3.4.1. Changes in the Appearance of Strawberries

**Figure 7:**
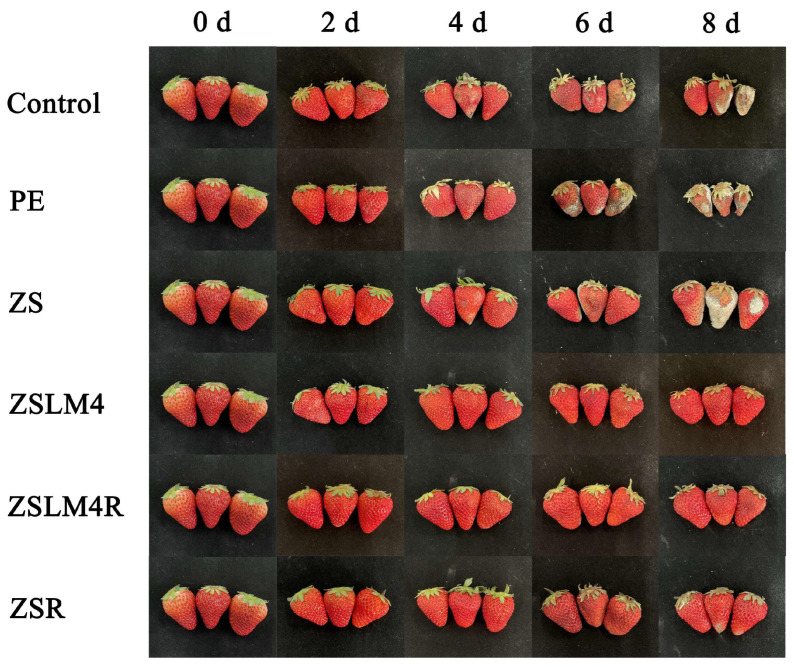
The appearance of strawberries packaged with zein/shellac composite films loaded with LM and RES.

To evaluate the preservation efficacy of the developed films, strawberries were selected as a popular but highly perishable fruit subject to microbial spoilage and quality deterioration. Fresh strawberries were packaged using various electrospun zein/shellac composite films, and unwrapped and commercial polyethylene (PE)-wrapped strawberries were used as the control. All samples were stored at 25 °C, and visual changes were monitored (Figure 7). As expected, both unwrapped and PE-wrapped strawberries exhibited rapid and severe decay and deterioration. Significant shrinkage, softening, decay, and visible mold development were apparent by day 4; clear signs of decay, deterioration, and mold were present by day 8. Similar results have been found in strawberries packaged with PE film [1]. This confirms that conventional PE film, lacking active properties, provides minimal protection against spoilage under these storage conditions. Zein/shellac composite film (ZS) showed the similar deterioration phenomenon by day 8. However, the strawberries packaged with ZS composite film loaded with LM/RES (ZSLM4, ZSLM4R, and ZSR) only displayed slight shrinkage and softening by day 8, with no significant decay, deterioration, or mold. This demonstrates that composite films loaded with LM and RES possess superior moisture and oxygen barrier properties, which slow moisture loss and inhibit respiration.

#### 3.4.2. Changes in the Weight Loss of Strawberries

**Figure 8:**
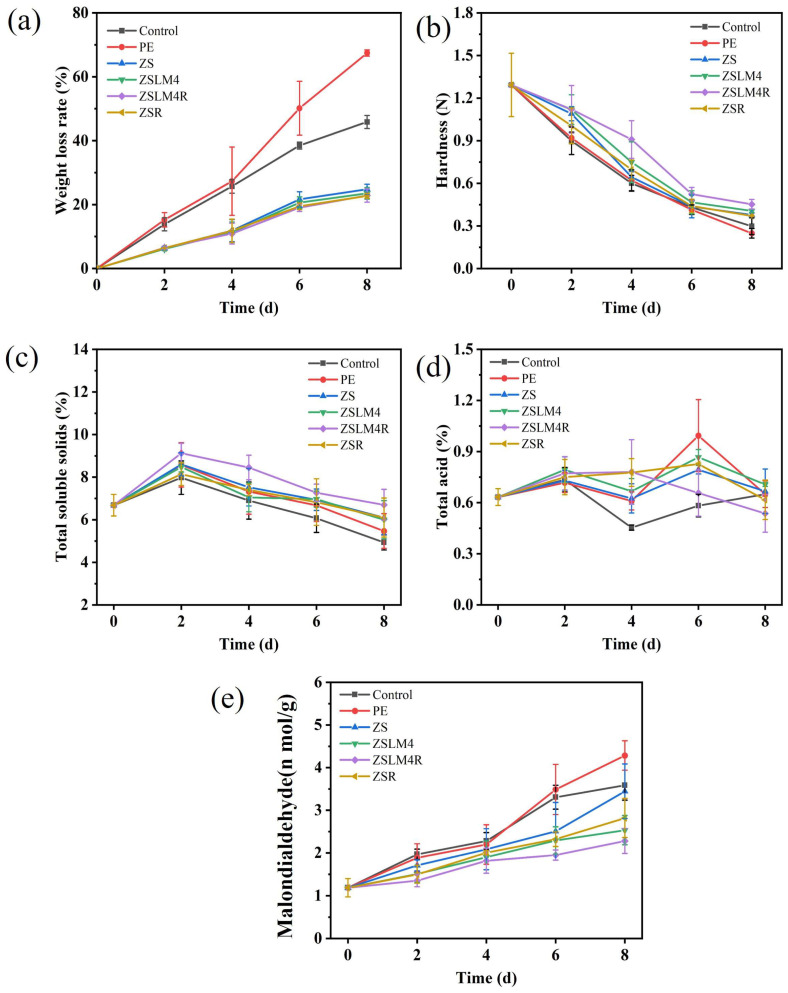
The influence of zein/shellac composite films loaded with LM and RES on strawberries: (**a**) weight loss; (**b**) hardness; (**c**) TSS; (**d**) TA; (**e**) MDA.

Weight loss rate is a critical indicator of both internal quality and appearance change for fruit during storage [43]. Given strawberries’ high moisture content (approximately 90%), the extent of moisture loss is used to assess the preservation effectiveness of ZS composite film loaded with LM/RES. As shown in Figure 8a, all strawberry samples exhibited a continuous increase in weight loss throughout the storage period at 25 °C. Though the rate of increase (slope) varied among the groups. Strawberries wrapped in commercial PE film suffered the most rapid and severe weight loss, reaching approximately 68% by day 8, which was consistent with the observed appearance of strawberries in Figure 7. Similarly, unwrapped (control) strawberries also suffered rapid and severe weight loss (approximately 46% by day 8) due to direct moisture release into the air. However, strawberries packaged with zein/shellac composite film loaded with LM/RES (ZSLM4, ZSLM4R, and ZSR) demonstrated a significant reduction in weight loss compared to the unwrapped and PE-wrapped controls; especially those wrapped in ZSLM4R composite film showed the least amount of weight loss (only 22.81%) throughout the storage time. This phenomenon is likely due to its superior barrier properties, which can significantly slow down water loss, and the film’s dense network structure also helps retain moisture, thereby prolonging the shelf life of the strawberries.

#### 3.4.3. Changes in the Hardness of Strawberries

Fruit firmness, represented by hardness, is a crucial quality parameter that directly impacts consumers’ acceptance. The changes in strawberry hardness over the 8-day storage period at 25 °C are presented in Figure 8b. As depicted, compared with the unpacking and PE-packed group of strawberries, incorporation of LM/RES active compounds into the zein/shellac films yielded a notable improvement in hardness retention; particularly, ZSLM4R film-coating treatment exhibited the highest hardness value (0.45 N) at the end of storage, which was significantly higher than the hardness value of other treatment groups (ZS 0.37 N; ZSLM4 0.40 N; ZSR 0.37 N). This is likely attributed to the fact that the zein/shellac composite film loaded with LM/RES effectively inhibited strawberry respiration and cell wall and cellulose degradation and maintained cellular integrity.

#### 3.4.4. Changes in the TSS and TA of Strawberries

Total soluble solids (TSSs) and titratable acidity (TA) are critical determinants of strawberry flavor, sweetness–sourness balance, and overall consumer acceptability. The changes in TSS of strawberries over storage time are shown in Figure 8c. All strawberry groups exhibited a similar trend: a slight initial increase (0–2 days) followed by a gradual decline. This phenomenon likely results from polysaccharide hydrolysis into monosaccharides during early ripening of strawberries (0–2 days), while subsequently, strawberries suffer from nutrient loss from respiratory metabolism and microbial activity during the storage period of 3–8 days [44]. Furthermore, strawberries packaged with the active films, particularly the dual-additive ZSLM4R film, demonstrated superior TSS retention. By day 8, the TSS content in the ZSLM4R group (6.7%) was significantly higher than all other treated groups; this indicated that the zein/shellac composite film loaded with LM/RES could effectively delay the soluble solids’ depletion and extend the strawberries’ shelf life.

TA is a crucial taste component of fruit and reflects the intrinsic acidity of the fruit. TA changes in the different groups are shown in Figure 8d. An initial rapid increase (0–2 days) was observed across all groups; this is likely owing to moisture loss and fungal proliferation producing specific compounds during storage [43]. Subsequently (2–4 days), TA values tended to decline slightly, as organic acids are utilized as respiratory substrates and potentially degraded by microbial activity. Notably, the ZSLM4R group maintained a high TA level throughout the later storage period (days 3–8) and displayed the slow rate of decline, which may be attributed to the fact that the composite film effectively inhibited the metabolic rate of strawberries and slowed down the consumption of TA.

#### 3.4.5. Changes in the MDA of Strawberries

Malondialdehyde (MDA) serves a critical role for evaluating the overall freshness and antioxidant status of fruits during storage [45]. As illustrated in Figure 8e, the MDA content in all strawberry groups exhibited a gradual initial increase during the first four days of storage at 25 °C, which indicates that the strawberries were in a relatively stable state with low oxidative stress during early storage. A significant change trend emerged after day 4; unpackaged and PE-wrapped strawberries displayed a sharp and substantial increase in MDA content. In contrast, the ZS, ZSLM4, ZSLM4R, and ZSR groups maintained lower MDA levels. Notably, strawberries packaged with the dual-additive composite film (ZSLM4R) exhibited the lowest MDA levels throughout the storage period, particularly after day 4, and showed the smallest overall increase. This highlights the contribution of the incorporated antioxidants LM and RES in the zein/shellac composite film, mitigating oxidative damage for strawberry preservation.

## 4. Conclusions

In this study, LM- and RES-loaded zein/shellac packaging films were fabricated via coaxial electrospinning and applied to strawberry packaging. Structural characterization revealed that LM/RES-loaded zein/shellac composite films (ZSLM4R) showed uniform and smooth morphology. The FTIR results showed that hydrogen bonding and electrostatic interactions between LM/RES and zein/shellac composite film stabilize the structure of ZSLM4R composite film. Enhanced mechanical strength and reduced water vapor permeability (WVP) were observed upon LM/RES incorporation. Furthermore, after the addition of LM and RES, the composite films showed excellent antioxidant capacity (DPPH/ABTS/FRAP assays) and antimicrobial properties against *E. coli* and *S. aureus*, with the ZELM4R composite film exhibiting relatively better performance. Additionally, strawberries packaged with LM/RES-loaded zein/shellac film were capable of extending the shelf life of strawberries and maintaining the quality of strawberries after harvest. In summary, the LM/RES-loaded zein/shellac packaging exhibited an excellent protection and preservation effect on strawberries, thus providing a new method for the preservation of perishable fruit.

## Figures and Tables

**Table 1:** The addition amounts of LM and RES in spinning solutions.

	LM (%; *w*/*w*)	RES (%; *w*/*w*)
ZS	0	0
ZSLM1	1	0
ZSLM2	2	0
ZSLM4	4	0
ZSLM4R	4	3
ZSR	0	3

## Data Availability

The original contributions presented in this study are included in the article. Further inquiries can be directed to the corresponding author.
